# Assessing soil degradation under land-use change: insight from soil erosion and soil aggregate stability in a small karst catchment in southwest China

**DOI:** 10.7717/peerj.8908

**Published:** 2020-04-06

**Authors:** Man Liu, Guilin Han

**Affiliations:** Institute of Earth Sciences, China University of Geosciences (Beijing), Beijing, China

**Keywords:** Soil aggregate stability, Soil erodibility, K factor, Soil degradation, Agricultural abandonment, Karst catchment, Southwest china

## Abstract

**Background:**

Soil erodibility (K factor) and soil aggregate stability are often used to assess soil degradation in an erodible environment. However, their applicability under land-use change is uncertain, especially agricultural abandonment.

**Methods:**

Different land-use types, including cropland, abandoned cropland, and native vegetation land, were converted into the successive stages following agricultural abandonment by space-for-time substitution approach in a small karst catchment, Southwest China. The indexes of soil aggregate stability and K factor of the Erosion Productivity Impact Calculator (EPIC) model in soil profiles were calculated to identify which method is suitable to indicate soil degradation under land-use change.

**Results:**

The indexes of soil aggregate stability in the soils at 0∼30 cm depth under native vegetation land were significantly larger than those under cropland and slightly larger than those under abandoned cropland. The K factor was not significantly different among the three land use examples because the EPIC model does not consider soil permeability. In the soil organic carbon (SOC)-rich soils (>2%), the K factor was significantly correlated with silt and clay content ranging within a narrow scope of near 0.010 t hm^2^ h/hm^2^/MJ/mm. While in the SOC-poor soils, the K factor was significantly increased with decreasing SOC content and was significantly correlated with soil aggregate stability.

**Conclusions:**

Soil aggregate stability is more suitable to indicate soil degradation under land-use change. Sufficient SOC in erodible soils would restrain soil degradation, while SOC loss can significantly increase soil erosion risk.

## Introduction

Soil erosion, which is one of the form of soil degradation, has seriously threatened environmental sustainability, economic development, and residential survival (Annabi et al., 2017; Ye et al., 2018). Soil erodibility is generally considered as an inherent soil property that the ease of soil detachment by rainfall and/or surface flow (Renard et al., 1997). In the Universal Soil Loss Equation (USLE) model, the concept of soil erodibility is introduced as the K factor, which is defined as the amount of soil loss caused by rainfall, runoff, or seepage within a standard unit based on substantial field data (Wischmeier & Smith, 1965), thus the K factor is also a key parameter to assess the susceptibility to soil erosion (Wang et al., 2013). Many nomographs of K factor are established based on their quantitative relationships with soil properties, including soil particle distribution, soil organic matter (SOM), soil permeability, and soil structure (Wang, Zheng & Guan, 2016), which also avoids the expensive observation periods of direct measurement. The K factor in the Erosion Productivity Impact Calculator (EPIC) model, which is calculated using the parameters of soil organic carbon (SOC) content and soil particle distribution, is widely used for the prediction of soil erosion on a catchment scale (Sharpley & Williams, 1990), as well as the indicator of soil degradation (Zhang et al., 2018).

In China, karst landscapes account for 13.5% of total land area (Wang, Liu & Zhang, 2004; Zhang et al., 2019b), where have also occurred many environment problems (Han et al., 2009; Han et al., 2019; Zeng et al., 2020). Ecologically fragile karst land under long-term irrational agricultural activities has occurred serious soil degradation, including soil erosion and nutrient loss (Liu et al., 2016). In the karst region of Southwest China, the researches of soil erodibility mainly focus on large catchment, mountain region, and slope farmland to illustrate the temporal and spatial variability of the K factor in different estimation models, as well as its influencing factors, including soil properties, geology, geomorphology, and agricultural management (Xu et al., 2008; Zeng et al., 2017). For example, Li et al. (2016) reported that soil erosion mainly occurred on the slope steepness within 8°∼25°; Yang et al. (2014) suggested that southeastern Guilin had a relatively high risk of soil erosion than the northwest; and Zeng et al. (2017) analyzed the temporal variability of soil erodibility in a mountainous area from 2000 to 2013, indicating that slight and middle erosion risk increased, while heavy erosion risk decreased. In the karst region, the K factor is commonly obtained based on the USLE model and GIS, direct measurement in the field is limited due to serious underground soil leaks (Zeng et al., 2017).

Soil aggregates are commonly regarded as the basic units of soil structure, which provide the space for the transports of soil water and dissolved nutrients, soil microbe activity, and root extension (Blanco-Canqui & Lal, 2004). Soil aggregate stability regarded as an important index of soil quality can be used for the indicator of soil degradation under land-use change (Ye et al., 2018), due to its rapid response to land management (Six, Elliott & Paustian, 2000; Choudhury et al., 2014). In a large catchment scale, the calculated K factor can be perfectly executed to indicate soil degradation, while it will be restricted in a small catchment scale because some soil properties may be constant. Thus, the results of soil aggregate stability can be used to check the applicability of the K factor to indicate soil degradation under land-use change in a small catchment.

For alleviating the problems of soil degradation, many low yielding and steeply sloping croplands have been abandoned and spontaneously recovered under the ‘Grain for Green Project (GGP)’ since the 1990s (Liu et al., 2019a). The assessment of soil degradation in the land restoration process is also important for the sustainable development of the living environment and agricultural production in the local area, but relevant works in the karst region have not been reported. Furthermore, in addition to surface soil erosion, underground soil leaks are also widely present (Zeng et al., 2017), thus soil structure and erodibility of the deep soil should be concerned. These evaluations of whole soil profiles are key to accurately predicting karst soil loss. In the present study, the researches of soil aggregate stability and the K factor in different land-use types, including cropland (CL), abandoned cropland (AL), and native vegetation land (NV), were converted into the studies along the successive stages following agricultural abandonment (land-use change) by the space-for-time substitution approach (Blois et al., 2013; Liu, Han & Zhang, 2020). Soil aggregate distribution, soil particle distribution, and SOC content were measured, then the indexes of soil aggregate stability and soil erodibility K factor were calculated in soil profiles under the three land types in a small karst catchment, Southwest China. The objectives of this study were: (1) to analyze the changes in soil aggregate stability and the K factor following agricultural abandonment; (2) to analyze the applicability of the two methods to indicate soil degradation in the small catchment under land-use change; and (3) to identify the key factor affected soil degradation in karst region.

## Materials & Methods

### Study area

**Figure 1:**
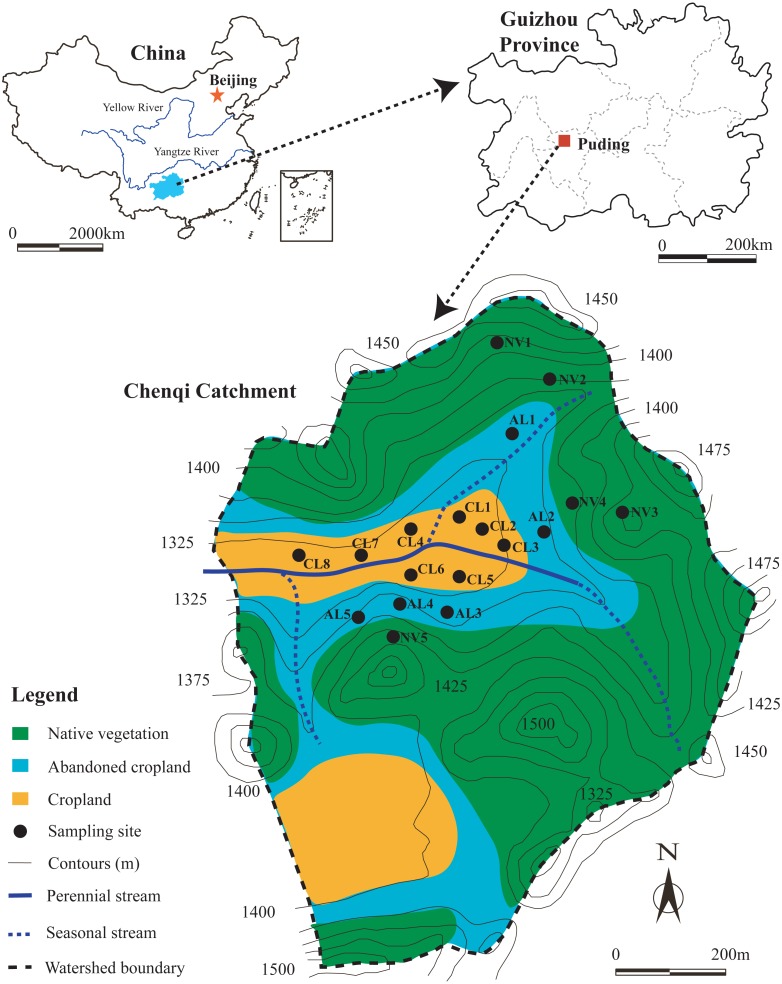
Location of study area and sampling sites.

The study sites were located in Chenqi catchment (26°15.779′–26°16.710′N, 105° 46.053′–105°46.839′E) with an area of 1.54 km^2^, at Puding county, Guizhou province, Southwest China (Fig. 1). The region controlled by sub-tropical monsoonal climate has an average annual temperature of 15.1 °C and an average annual precipitation of 1400 mm (Han, Li & Tang, 2017). The calcareous soils, which are Mollic Inceptisols in the Soil Taxonomy of United States Department of Agriculture (USDA), are developed from limestone, and soil thickness ranges from 10 to 160 cm (Zhang et al., 2019a). In the study area, much soil is unevenly distributed in the crevices between rocks, which is translocated from topsoil at upslope. Native vegetation lands (NV) are mainly distributed on the hilltop and hillside (Fig. 1), these are covered by mixed evergreen and broadleaved deciduous forest or shrub-grass. Abandoned croplands (AL) are mainly located at the bottom of hillside, these have been developed from croplands and pear orchards in 8 years ago, and have evolved into shrublands or grasslands at present. Croplands are located in the depression of this catchment, these are characterized by terraced fields, long-term tillage, and alternate planting of corn and rice (Liu, Han & Zhang, 2020). Croplands are mainly fertilized by urea and manure from May to August (Zeng et al., 2019).

**Table 1:** Visible characteristics of the soil profiles.

Sampling site	Elevation (m)	Soil profile thickness (cm)	Visible characteristics
Native vegetation (NV)			
NV1	1,420 m	30 cm	0∼30 cm: Black-brown, fine grained, loose, abundant plant roots and debris, accumulated on the unweathered rock
NV2	1,375 m	90 cm	0∼50 cm: Black-brown, fine grained, loose, abundant plant roots and debris
			50∼90 cm: Red, clayey, tight, no rootlet
NV3	1,449 m	50 cm	0∼30 cm: Black-brown, fine grained, loose, abundant plant roots and debris
			30∼50 cm: Red, clayey, tight, no rootlet
NV4	1,370 m	50 cm	0∼50 cm: Black-brown, fine grained, loose, abundant plant roots and debris, accumulated in crevices between unweathered rocks
NV5	1,410 m	90 cm	0∼30 cm: Black-brown, fine grained, loose, abundant plant roots and debris
			30∼90 cm: Gray, clayey, tight, no rootlet
Abandoned cropland (AL)			
AL1	1,340 m	90 cm	0∼10 cm: Black-brown, fine grained, loose, abundant plant roots and debris
			10∼90 cm: Red, block, tight, few plant roots
AL2	1,345 m	90 cm	0∼10 cm: Brown, fine grained, loose, abundant plant roots and debris
			10∼90 cm: Yellow, block, tight, few plant roots
AL3	1,340 m	70 cm	0∼70 cm: Black-brown, fine grained, loose, abundant plant roots and debris, accumulated in crevices between unweathered rocks
AL4	1,361 m	70 cm	0∼20 cm: Black-brown, fine grained, loose, abundant plant roots and debris
			20∼70 cm: Red, block, tight, few plant roots
AL5	1,352 m	70 cm	0∼30 cm: Black-brown, fine grained, loose, abundant plant roots and debris
			30∼70 cm: Red, block, tight, few plant roots
Cropland (CL)			
CL1	1,315 m	30 cm	0∼10 cm: Brown, fine grained, loose, few plant roots
			10∼30 cm: Yellow, block, tight, no rootlet
CL2	1,319 m	70 cm	0∼10 cm: Brown, fine grained, loose, few plant roots
			10∼70 cm: Yellow, block, tight, no rootlet
CL3	1,325 m	50 cm	0∼50 cm: Brown, fine grained, loose, few plant roots accumulated on the unweathered rock
CL4	1,320 m	50 cm	0∼50 cm: Brown, fine grained, loose, few plant roots accumulated on the unweathered rock
CL5	1,320 m	70 cm	0∼70 cm: Brown, fine grained, loose, few plant roots, accumulated on the unweathered rock
CL6	1,321 m	50 cm	0∼50 cm: Brown, fine grained, loose, few plant roots, accumulated on the unweathered rock
CL7	1,321 m	70 cm	0∼10 cm: Brown, fine grained, loose, few plant roots
			10∼70 cm: Red, block, tight, no rootlet
CL8	1,322 m	30 cm	0∼10 cm: Brown, fine grained, loose, few plant roots
			10∼30 cm: Yellow, block, tight, no rootlet

### Soil sampling

The hills, which are considered most likely to erode, are mainly located in the middle to north of this catchment. Moreover, cropland, abandoned cropland, and native vegetation land are widely distributed in this region. Thus, soil sampling sites in this region of the catchment adequately support the studies of soil degradation at different stages following agricultural abandonment. In the study area, the native vegetation lands are public areas. Soil sampling in croplands and abandoned croplands were conducted after receiving a permission from the farmer, Aihua Li. Total 18 soil profiles in the study area, 8 soil profiles under cropland (CL), 5 soil profiles under abandoned cropland (AL), and 5 soil profiles under native vegetation land (NV) were selected, location and vertical characteristic of these soil profiles are shown in Fig. 1 and Table 1. About 1 × 0.5 m pit was dug by a shovel, and chose a side of the pit as the soil profile for collecting soil samples. The thicknesses of these soil profiles ranged from 30 cm to 90 cm depended on bedrock depth. Soil samples were collected from 0∼10, 10∼20, 20∼30, 30∼50, 50∼70, and 70∼90 cm depth, the number of samples collected at different profiles was 3 to 6, with the total number of 83 soil samples. After the soil samples were collected, the pits were refilled by the original soils and marked by red flag to hint the subsequent sampler. In the small catchment, parent material and climate at all sampling sites are same. Croplands and abandoned croplands were belong to terraced fields without slope length and slope steepness. And the sites of native vegetation lands were selected the flat ground (slope steepness <5°). Thus the topography factor at all sites can be considered no difference. In the present study, differences in soil aggregate stability and soil erodibility in the soils at this three land-use types can be considered only to depend on land-use change.

### Soil analysis

Soil samples were air-dried at room temperature after removing big roots and stones. Different-sized aggregates were separated by the modified method of wet sieving (Liu, Han & Zhang, 2019). Macro-aggregates (250∼2,000 µm) and micro-aggregates (53∼250 µm) were collected after passing through 2000, 250, and 53 µm sifters; and silt + clay-sized fractions (<53 µm) were isolated using centrifugation (Liu et al., 2019b). All aggregates were dried at 55 °C until constant weight, then weighed. Macro-aggregates obtained through wet sieving are also water-stable aggregates, their mass percentage can be regarded as an index of soil aggregate stability. Furthermore, water shock for soil aggregates in the processes of wet sieving simulates the shock by runoff and raindrops. Thus the percent of water-stable macro-aggregates not only indicates soil structure, but also associates with soil erodibility.

For dispersing soil particles, organic bonding agents between soil mineral particles were removed using 10% hydrogen peroxide (H_2_O_2_) and calcareous cements were removed using 2 mol/L hydrochloric acid (HCl) (Liu, Han & Zhang, 2020). Soil particle distribution was measured by the laser particle size analyzer (Mastersizer 2000, Malvern, England) with a precision of ± 1% (equivalent volume proportion), in the Center Laboratory for Physical and Chemical Analysis, Institute of Geographic Sciences and Natural Resources Research, Chinese Academy of Sciences.

Dried soil samples were ground using agate mortar and passed through 150 µm steel sifter. The carbonates in pulverous soil samples were removed using 0.5 mol/L HCl for 24 h, then washed with deionized water until neutral supernatant (Liu, Han & Zhang, 2020). Treated soil samples were dried at 55 °C until constant weight, then weighed and ground into powder (<150 µm). The C content was analyzed using combustion method in an elemental analyzer (Elementar, Vario TOC cube, Germany) in the Laboratory of Surficial Environment Geochemistry, China University of Geosciences (Beijing). The reproducibility was determined through replicate measurements of standard material low organic soil (OAS-B2152, C content: 1.55 ± 0.06%), which was better than ± 0.1%. Actual SOC content can be obtained by multiplying the measured C content by the rate of soil sample mass after carbonate loss to the mass before carbonate loss.

### Calculation of the indexes of soil aggregate stability

Water-stable macro-aggregates (WSMacA) are the summation of 250∼2,000 µm sized aggregates using wet sieving method.

(1) \begin{eqnarray*}\text{WSMacA}(\text{%})=\text{percentage of macro-aggregates}.\end{eqnarray*}

Mean weight diameter (MWD) and geometric mean diameter (GMD) of soil aggregates are calculated as (Choudhury et al., 2014):

(2) \begin{eqnarray*}\mathrm{MWD}(\mathrm{mm})& =\sum _{\mathrm{k}=1}^{\mathrm{n}}{\mathrm{X}}_{\mathrm{ k}} \times {\mathrm{M}}_{\mathrm{k}}\end{eqnarray*}

(3) \begin{eqnarray*}\mathrm{GMD}(\mathrm{mm})& =\exp \nolimits \sum _{\mathrm{k}=1}^{\mathrm{n}}\ln \nolimits ({\mathrm{X}}_{\mathrm{ k}})\times {\mathrm{M}}_{\mathrm{k}}\end{eqnarray*}

where K is the size-class aggregates (*k* = 1, 2, 3 indicate macro-aggregates, micro-aggregates, and silt + clay sized fractions, respectively); X_k_ (mm) is the mean diameter of the size-class aggregates (X_1_ = $ \frac{(250+2000)}{2000} ,{\mathrm{X}}_{2}= \frac{(53+250)}{2000} ,{\mathrm{X}}_{3}= \frac{53}{2000} $); and M_k_ (%) is the percentage of the size-class aggregates.

The aggregate ratio (AR) of soils is calculated as:

(4) \begin{eqnarray*}\mathrm{AR}= \frac{\text{percentage of macro-aggregates}}{\text{percentage of micro-aggregates}} .\end{eqnarray*}

### Calculation of K factor

Soil erodibility factor in EPIC model (K_epic_) is calculated as (Sharpley & Williams, 1990):

(5) \begin{eqnarray*}\text{Kepic}& = \left\{ 0.2+0.3\exp \nolimits \left[ - 0.0256\mathrm{S} \left( 1- \frac{\mathrm{F}}{100} \right) \right] \right\} { \left( \frac{\mathrm{F}}{\mathrm{M}+\mathrm{F}} \right) }^{0.3}\nonumber\\\displaystyle & \left[ 1.0- \frac{0.25\mathrm{C}}{\mathrm{C}+\exp \nolimits \left( 3.72-2.95\mathrm{C} \right) } \right] \left[ 1.0- \frac{0.7\mathrm{E}}{\mathrm{E}+\exp \nolimits \left( -5.51+22.9\mathrm{E} \right) } \right] \end{eqnarray*}

where S, F, M (%) represent the percentage of sand (0.05∼2.0 mm), silt (0.002∼0.05 mm), and clay (<0.002 mm), respectively, according to the soil texture classification of the USDA; C (%) is the SOC content; E = 1–S/100. The result is expressed in the American unit (t acre h/100 acre/ft/tanf/in), then it should be translated into the international unit (t hm^2^ h/hm^2^/MJ/mm) by multiplying 0.1317.

To make the K factor respect to Chinese soils, the K_epic_ factor is calibrated as the formula (Zhang et al., 2008):

(6) \begin{eqnarray*}\mathrm{K}=-0.01383+0.5158~\text{Kepic}.\end{eqnarray*}

### Statistical analysis

One-way ANOVA with least significant difference (LSD) test was performed to determine the differences in soil particle distribution, SOC content, K factor, and soil aggregate stability indexes among different land-use types or among different soil depths at the threshold of the level of *P* <0.05. Two-way ANOVA analysis with LSD test was used to examine the significance of land-use type, soil depth, and their interactions on soil aggregate stability indexes, soil particle distribution, SOC content, and the K factor. The relationships between K factor and the soil properties, including SOC content and soil particle distribution, were determined by line regression analysis, and coefficient *R* ^2^ and *P*-value showed the fitting degree of the best-fit regression line. Pearson’s correlation analysis was used to analyze the relationships between soil aggregate stability indexes and K factor, soil particle distribution, and SOC content.

Principle component analysis (PCA) was used to transform the original soil physiochemical properties and indexes that can indicate soil degradation degree into two component compound variables, and each compound variable was uncorrelated with all other compound variables. The principle component whose eigenvalue was exceeding 1 would be extracted, and component matrix, eigenvalues, variance, and cumulative variance were exhibited. The figures and statistical analyses were done using SigmaPlot 12.5 (Systat Software GmbH, Erkrath, Germany) and SPSS 18.0 (SPSS Inc., Chicago, IL, USA) software package, respectively.

## Results

### Soil properties, soil aggregate stability and soil erodibility K factor

In the study area, clay contents in the soil profiles of all sampling sites ranged from 12% to 25%, silt contents ranged from 75% to 88%, while the soils seldom contained sand sized particles (Table S1). The SOC contents ranged from 0.33% to 5.80%, exhibited a decreasing trend with the increase of soil depth. The proportions of macro-aggregates, micro-aggregates, and silt + clay sized fractions in all soils varied within the wide range of 23%∼91%, 5%∼24%, and 4%∼61%, respectively. Generally, macro-aggregate proportions decreased with increasing soil depth, while the proportions of micro-aggregates and silt + clay sized fractions increased. The soil aggregate stability index of WSMacA equals macro-aggregate proportion, thus WSMacA also ranged from 23% to 91%. Other indexes, including MWD, GMD, and AR, had the range of 0.302∼1.030 mm, 0.084∼0.868 mm, and 1.46∼19.53, respectively. The changes in these indexes along soil profiles were similar to the trend of macro-aggregate proportion with depth, due to the large weight of macro-aggregate in the calculation of these indexes. Soil erodibility K factor in these profiles at all depths ranged from 0.00956 to 0.01838, with an increasing trend from the top to the bottom of the profile.

### Effects of land-use type and soil depth on soil aggregate stability and soil erodibility K factor

**Figure 2:**
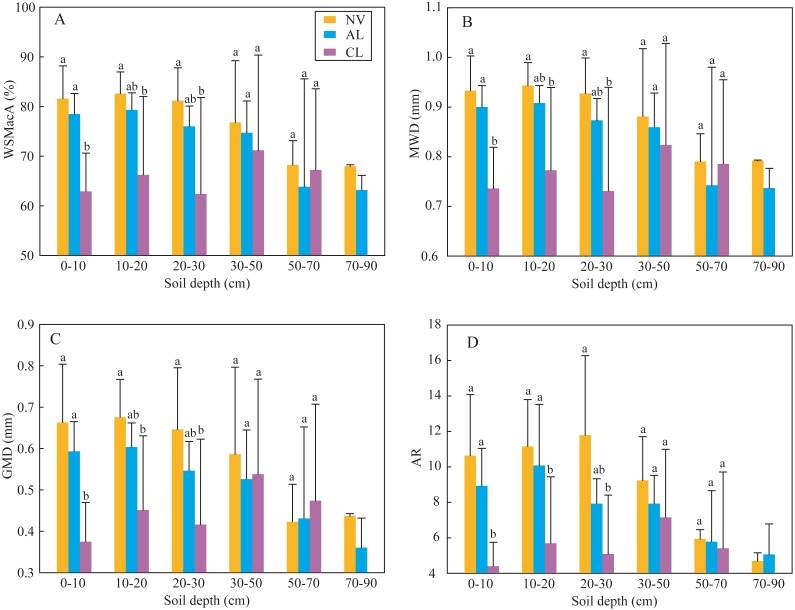
Soil aggregate stability indexes of WAMacA (A), MWD (B), GMD (C) and AR (D) in different depths of soil layer at different stages after agricultural abandonment. Lowercases indicate significant differences of these soil aggregate stability indexes among the different stages after agricultural abandonment at the threshold of *P* < 0.05 level, based on the least significant difference (LSD) test. WAMacA, water-stable macro-aggregate; MWD, mean weight diameter; GMD, geometric mean diameter; AR, the ratio of macro-aggregate to micro-aggregate; CL, cropland; AL, abandoned cropland; and NV, native vegetation land.

**Figure 3:**
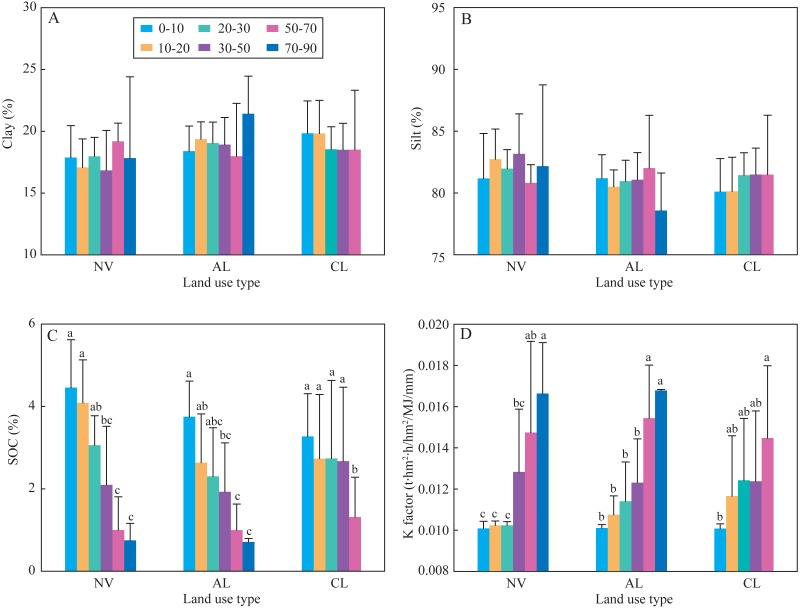
Clay content (A), silt content (B), SOC content (C), and the K factor (D) under the three land use types at different soil depths. Lowercases indicate significant differences of SOC content and the K factor among the different depths at the threshold of *P* < 0.05 level, based on the least significant difference (LSD) test. Clay and silt content are not significantly different among the different depths, thus the lowercase is not labeled. CL, cropland; AL, abandoned cropland; and NV, native vegetation land.

The results of indexes of two-way ANOVA analysis (Table 2) showed that the four indexes of soil aggregate stability (WSMacA, MWD, GMD, and AR) were affected by land-use types significantly; soil depth did not significantly affect them, except AR. Soil erodibility K factor and SOC content were only significantly affected by soil depth. Soil particle distribution was not affected by both land-use type and soil depth, which showed constant composition with depth at different sites within this small catchment. Based on the above analysis, one-way ANOVA analysis was used to determine the significance of land-use type on soil aggregate stability at different depths (Fig. 2) and the significance of soil depth on SOC content and K factor under different land-use types (Fig. 3). In the soils at 0∼30 cm depth, the four indexes of soil aggregate stability in the soils under native vegetation land were significantly higher than these under cropland (Fig. 2). While the four indexes under native vegetation land were not significantly difference with these under abandoned cropland. However, the four indexes among the three land-use types were not significantly different in the soils below 30 cm. Clay contents and slit contents were almost constant among different land-use types and different depths (Fig. 3). In the soils under native vegetation land and abandoned cropland, the SOC contents significantly decreased with increasing depth, while the K factors significantly increased. However, the SOC contents and K factors under cropland did not significantly vary with increasing depth in the soils at 0∼50 cm depth.

**Table 2:** Effects of land use type, soil depth, and their interactions on soil aggregate stability indexes, soil particle distribution, SOC content, and the K factor. Two-way ANOVA analysis with LSD test; “ns” means no-significant differences.

Factor	Variable	Type III sum of squares	df	Mean square	*F*	Sig.
Soil depth	WSMacA	1035.380	5	207.076	1.324	0.265 ns
	MWD	0.114	5	0.023	1.271	0.287 ns
	GMD	0.225	5	0.045	1.840	0.117 ns
	AR	129.819	5	25.964	2.891	0.020
	SOC	65.660	5	13.132	8.048	0.000
	Clay	10.926	5	2.185	0.312	0.904 ns
	Silt	15.309	5	3.062	0.410	0.840 ns
	K factor	0.000	5	0.000	10.369	0.000
Land use type	WSMacA	1,894.041	2	947.021	6.056	0.004
	MWD	0.202	2	0.101	5.616	0.006
	GMD	0.267	2	0.134	5.468	0.006
	AR	206.646	2	103.323	11.505	0.000
	SOC	2.960	2	1.480	0.907	0.409 ns
	Clay	29.077	2	14.539	2.077	0.133 ns
	Silt	23.003	2	11.501	1.539	0.222 ns
	K factor	0.000	2	0.000	0.358	0.700 ns
Soil depth × Land use type	WSMacA	806.588	9	89.621	0.573	0.814 ns
	MWD	0.094	9	0.010	0.578	0.811 ns
	GMD	0.199	9	0.022	0.904	0.527 ns
	AR	80.510	9	8.946	0.996	0.452 ns
	SOC	8.795	9	0.977	0.599	0.793 ns
	Clay	28.721	9	3.191	0.456	0.899 ns
	Silt	27.619	9	3.069	0.411	0.925 ns
	K factor	0.000	9	0.000	0.384	0.939 ns

### Indication of soil degradation by soil aggregate stability and K factor

**Figure 4:**
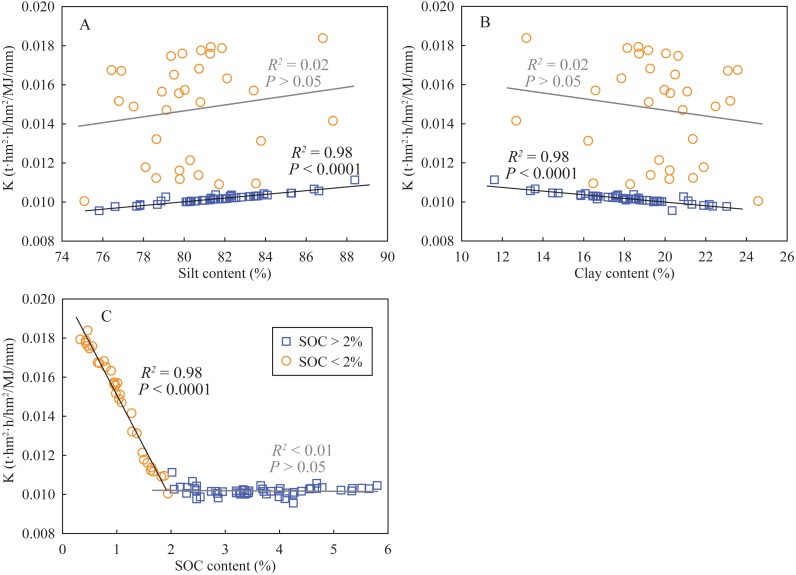
Relationships between K factor and silt content (A), clay content (B), and SOC content (C).

According to the results of PCA, two principal components (PC) were extracted (Table 3). The PC1 explained 62.99% of total variance and predominantly included the four indexes of soil aggregate stability (WSMacA, MWD, GMD, and AR), SOC content, and K factor; the PC2 explained 20.59% of total variance with significant loadings of clay and silt content. The Component matrix of these variables showed that soil aggregate stability (0.94) is a better indicator of soil degradation rather than the K factor (0.74) in this study area. Moreover, the coefficients of SOC, K factor, clay and sand are 0.724, −0.737, −0.551 and 0.510, respectively, which indicates that the K factor exhibits a stronger correlation SOC content compared to clay and silt content. Subsequently, line regression analyses were used to determine the relationships between the K factor and SOC content, silt content, and clay content (Fig. 4). The K factor in the soils that SOC contents were larger than 2% fluctuated within a small range near 0.010, while significantly increased with decreasing SOC contents when SOC contents were lower than 2%. Similarly, in the soils of >2% SOC content, the K factors significantly correlated with silt contents (positively) and clay contents (negatively). However, the correlations between the K factors and soil particle distribution were not observed in the soils that SOC contents were lower than 2%. These results indicated that the estimation of the K factor in the SOC-rich soils mainly depended on soil particle distribution, while in it mainly depended on SOC content. The results from Pearson’s correlation analysis (Table 4) showed that the four indexes of soil aggregate stability were significantly correlated with silt contents (positive) and clay contents (negative) in the SOC-rich soils (>2%); while the SOC-poor soils (<2%), they exhibited significant positive correlations with SOC contents and negative correlations with K factors. These results showed that soil aggregate stability could be used to indicate the degree of soil degradation in the SOC-rich soils; while both soil aggregate stability and the K factor were feasible in the SOC-poor soils.

**Table 3:** Principal component analysis of soil aggregate stability indexes, soil particle distribution, SOC content, and K factor.

Variable	Component	
	PC1	PC2
WSMacA	0.940	0.184
MWD	0.939	0.180
GMD	0.963	0.084
AR	0.846	0.158
SOC	0.724	0.098
Clay	−0.551	0.828
Silt	0.510	−0.852
K factor	−0.737	−0.358
Eigenvalues	5.039	1.647
Variance (%)	62.993	20.592
Cumulative (%)	62.993	83.584

**Table 4:** Pearson’s correlation analysis between soil aggregate stability indexes and the K factor, silt, clay and SOC contents.

	WSMacA	MWD	GMD	AR
SOC >2%				
Silt	0.30^*^	0.31^*^	0.37^**^	0.24
Clay	−0.36^**^	−0.37^**^	−0.43^**^	−0.28^*^
SOC	0.13	0.14	0.18	−0.01
K factor	0.21	0.21	0.27	0.20
SOC <2%				
Silt	0.14	0.14	0.25	0.05
Clay	−0.14	–0.14	−0.25	−0.05
SOC	0.64^**^	0.64^**^	0.67^**^	0.54^**^
K factor	−0.59^**^	–0.59^**^	−0.62^**^	−0.54^**^

**Notes.**

* *P* < 0.05.

** *P* < 0.01.

## Discussion

### Effects of land-use change on soil aggregate stability

Soil aggregate stability generally attributes to frequent soil microbial activities and abundant SOM in surface soils (Bach & Hofmockel, 2014). Soil organic matters, those are the organic binding agents derived from plant debris, root exudates, and microbial secretions, can significantly affects soil aggregate stability in C-rich soils (Six et al., 2004). In the present study, soil aggregate stability in the soils under cropland was significantly lower than that under abandoned cropland and native vegetation land (Fig. 2), while SOC contents were not significantly different among the three land types (Fig. 3 and Table 2). This result infers that soil aggregate stability is also affected by other factors. In addition to organic binding agents, soil aggregate stability is also affected by inorganic binding agents, i.e., metal cations, including Ca^2+^ and Mg^2+^ (Six et al., 2004). Jiang et al. (2006) reported that long-term tillage significantly reduced Ca^2+^ and Mg^2+^ contents in the karst soils. Thus low soil aggregate stability under cropland likely results from the loss of those metal cations. Land management (especially tillage) can also strongly affect soil aggregate stability. Long-term tillage influences the formation and turnover of water-stable macro-aggregates, i.e., WSMacA as one of soil aggregate stability indexes (Six, Elliott & Paustian, 2000; Choudhury et al., 2014). Moreover, macro-aggregates (>70%) generally accounted for the largest proportion in all sized aggregates (Table 2). Due to the large weight of macro-aggregate proportion in the calculation of MWD, GMD, and AR, thus the four indexes of soil aggregate stability show similar trends among the three land-use types as WSMacA. Overall, macro-aggregate plays an important role in affecting soil aggregate stability under land-use change. Soil aggregate stability can quickly respond to land-use change, resulting from the sensitive macro-aggregates to land management. However, reduced soil disturbance in abandoned cropland enhances the formation of water-stable macro-aggregates and soil aggregate stability. Vegetation restoration after agricultural abandonment generally enhances plant biomass, as well as soil microbial activity (Chavarria et al., 2018). The increases in plant biomass and soil microbial activity under abandoned cropland enhance the organic puts into the soils through litter, root exudates and microbial secretions (Liu, Han & Zhang, 2019), which increases soil aggregate stability after agricultural abandonment. These results indicated that soil aggregate stability significantly decreased under long-term tillage, and it could be recovered after 8 years agricultural abandonment.

### Limitation in soil erodibility K factor of the EPIC model in karst soils

Soil erodibility K factor of EPIC model was significantly correlated with soil particle distribution in the SOC-rich soils, while it was significantly correlated with SOC content in the SOC-poor soils (Fig. 4). The effects of soil particle on erodibility mainly depend on: (1) fine particles are more easily migrated by runoff, and (2) high soil permeability in coarse texture soils reduces water erosion (Ostovari et al., 2018). Soil organic matters, which act as matrixes to absorb soil water, improve anti-erosion ability through decreasing detachment of soil particles by runoff and raindrops and increasing soil shear strength (Zhu et al., 2010). They also bind soil particles to reduce the migration of fine particles through the formation of soil aggregates and organic–inorganic complexes, for example, allophane–Fe (Al)–OC (Six et al., 2004; Rodríguez et al., 2006; Wang, Fang & Chen, 2017). Furthermore, these soil aggregates and organic–inorganic complexes increase the sizes of matrix particles and soil pores, which enhances soil permeability. However, there was not reasonable that the K factor was not significantly different under land-use change (Table 2). A key limitation in soil erodibility of the EPIC model in karst soils is that it does not consider soil permeability associated with abundant aggregates. Vaezi et al. (2008) reported that calcium in calcareous soils strongly affected aggregate formation and soil structure and hence influenced soil erodibility. The second limitation in the K factor is the number of sites, which is associated with this catchment size. This defect is exhibited from the relationships between the K factor and soil particle distribution, i.e., the K factor only varied by less 0.001 with soil particle distributions in the SOC-rich soils due to constant silt and clay contents in this small catchment. A larger change in soil particle distribution for whole karst soils, thus the relationships between the K factor and soil particle distribution are feeble to generalize in whole karst region only based on the present study, widely sampling is necessary for the future work. The third limitation in the estimated K factor is the lack of contrast with the values derived from field measurement, which makes it impossible to quantify the importance of soil aggregate stability in estimating K factor. However, from the qualitative analysis, it can be convinced that soil permeability associated with soil aggregate stability plays an important role in estimating K factor in the karst soils.

### Indicator of land degradation in karst soils

Soil aggregate stability and soil erodibility are important indicators of soil degradation (Adhikary et al., 2014; Ye et al., 2018). According to the results of PCA, soil aggregate stability in SOC-rich soils is more suitable to indicate soil degradation than the K factor, mainly resulting from sufficient consideration in soil permeability and quickly responding to land-use change. For the low SOC soils (<2%) generally located at deep depth, soil degradation, including soil erodibility and soil structure, is also necessary to study due to the presence of underground soil leaks (Zeng et al., 2017). In the SOC-poor soils, SOC content exhibited a positive correlation with soil aggregate stability and showed a negative correlation with K factor (Fig. 4 and Table 4), suggesting that SOM played a key role in soil aggregate stability and soil erodibility, hence affected soil degradation. Soil organic matters can directly affect soil degradation due to the supply of mineral nutrients in their decomposition processes (Zhou et al., 2019). They also indirectly affect soil degradation through: (1) soil microbial richness, which is generally associated with SOM as the energy source for microbial growth (Zhu et al., 2010; Yang et al., 2018); (2) soil structure maintaining by the formation of soil aggregates (Six et al., 2004) and organic–inorganic complexes (Rodríguez et al., 2006; Wang, Fang & Chen, 2017). Moreover, the K factor remained relatively low value (near 0.010) when SOC content was larger than 2%; while it significantly increased when SOC content decreased below 2% (Fig. 4). Thus, the threshold value of SOC content is determined as 2%, decrease in SOC can significantly lead to soil degradation.

## Conclusions

Soil aggregate stability in the soils at 0∼30 cm depth under native vegetation land was significantly higher than that under cropland and slightly higher than that under abandoned cropland. Soil erodibility K factor was significantly increased with increasing depth, while SOC content was significantly decreased. Soil erodibility in the SOC-rich soils was mainly controlled by soil particle distribution, while it in the SOC-poor soils was mainly determined by SOM.

Soil aggregate stability is more suitable to indicate soil degradation under land-use change because the K factor of the EPIC model do not consider soil permeability. Sufficient SOC was underscored in restraining soil degradation based on the significant correlations between SOC content and soil aggregate stability, K factor in the SOC-poor soils.

##  Supplemental Information

10.7717/peerj.8908/supp-1 File S1 Raw data

10.7717/peerj.8908/supp-2 Table S1 Properties of all soil samples

## Additional Information and Declarations

### Data Availability

The following information was supplied regarding data availability: The raw measurements are available in the Supplemental Files.
