# Supplementary material for: Assessing soil degradation under land-use change: insight from soil erosion and soil aggregate stability in a small karst catchment in southwest China
Source: PeerJ. 2020 Apr 6;8:e8908. doi: 10.7717/peerj.8908 (PMC7144589; doi:10.7717/peerj.8908)
Supplement: Table S1 [file peerj-08-8908-s002.docx]

| **Site** | **Depth** | **Macro-aggregate** | **Micro-aggregate** | **Silt + clay size fraction** | **MWD (mm)** | **GMD**  **(mm)** | **AR** | **SOC**  **(%)** | **Clay**  **(%)** | **Silt (%)** | **Sand (%)** | **K factor** |
| --- | --- | --- | --- | --- | --- | --- | --- | --- | --- | --- | --- | --- |
| NV1 | 0-10 | 83.30 | 7.94 | 8.76 | 0.951 | 0.691 | 10.50 | 4.25 | 20.35 | 75.82 | 3.83 | 0.009558 |
| NV1 | 10-20 | 83.10 | 8.11 | 8.79 | 0.949 | 0.688 | 10.25 | 3.26 | 18.08 | 80.99 | 0.93 | 0.010091 |
| NV1 | 20-30 | 83.69 | 7.52 | 8.79 | 0.955 | 0.696 | 11.13 | 3.69 | 16.64 | 83.20 | 0.17 | 0.01028 |
| NV2 | 0-10 | 80.70 | 8.84 | 10.46 | 0.924 | 0.637 | 9.13 | 3.28 | 19.84 | 80.16 | 0.00 | 0.010015 |
| NV2 | 10-20 | 75.12 | 9.49 | 15.39 | 0.864 | 0.522 | 7.92 | 3.40 | 19.44 | 80.56 | 0.00 | 0.010049 |
| NV2 | 20-30 | 79.16 | 8.42 | 12.43 | 0.907 | 0.596 | 9.41 | 3.20 | 19.12 | 80.88 | 0.00 | 0.010081 |
| NV2 | 30-50 | 84.72 | 6.88 | 8.39 | 0.966 | 0.715 | 12.31 | 2.30 | 17.67 | 82.33 | 0.00 | 0.010362 |
| NV2 | 50-70 | 71.70 | 12.88 | 15.42 | 0.830 | 0.487 | 5.57 | 1.57 | 20.23 | 79.77 | 0.00 | 0.011613 |
| NV2 | 70-90 | 67.82 | 15.51 | 16.67 | 0.791 | 0.441 | 4.37 | 1.04 | 22.48 | 77.52 | 0.00 | 0.014886 |
| NV3 | 0-10 | 81.50 | 7.81 | 10.68 | 0.932 | 0.644 | 10.43 | 5.52 | 15.86 | 83.65 | 0.49 | 0.010321 |
| NV3 | 10-20 | 85.03 | 8.61 | 6.37 | 0.971 | 0.746 | 9.88 | 4.68 | 13.38 | 86.62 | 0.00 | 0.01057 |
| NV3 | 20-30 | 90.83 | 4.65 | 4.51 | 1.030 | 0.865 | 19.53 | 2.47 | 16.08 | 83.92 | 0.00 | 0.01043 |
| NV3 | 30-50 | 81.15 | 10.86 | 7.98 | 0.932 | 0.671 | 7.47 | 1.27 | 12.68 | 87.32 | 0.00 | 0.014151 |
| NV4 | 0-10 | 72.07 | 10.43 | 17.51 | 0.831 | 0.474 | 6.91 | 3.43 | 18.87 | 81.01 | 0.12 | 0.010089 |
| NV4 | 10-20 | 86.20 | 6.04 | 7.76 | 0.981 | 0.745 | 14.26 | 3.44 | 17.90 | 82.11 | 0.00 | 0.010185 |
| NV4 | 20-30 | 79.36 | 9.72 | 10.92 | 0.910 | 0.615 | 8.16 | 3.76 | 19.57 | 80.32 | 0.11 | 0.010024 |
| NV4 | 30-50 | 83.05 | 8.19 | 8.76 | 0.949 | 0.687 | 10.14 | 4.01 | 16.49 | 83.52 | 0.00 | 0.010305 |
| NV5 | 0-10 | 90.47 | 5.58 | 3.95 | 1.027 | 0.868 | 16.21 | 5.80 | 14.46 | 85.28 | 0.25 | 0.010461 |
| NV5 | 10-20 | 83.56 | 6.20 | 10.24 | 0.952 | 0.677 | 13.47 | 5.65 | 16.59 | 83.41 | 0.00 | 0.010295 |
| NV5 | 20-30 | 72.95 | 6.80 | 20.25 | 0.836 | 0.459 | 10.73 | 2.17 | 18.45 | 81.55 | 0.00 | 0.010377 |
| NV5 | 30-50 | 58.28 | 8.27 | 33.44 | 0.677 | 0.272 | 7.05 | 0.80 | 20.49 | 79.51 | 0.00 | 0.016512 |
| NV5 | 50-70 | 64.79 | 10.26 | 24.95 | 0.751 | 0.359 | 6.31 | 0.43 | 18.14 | 81.86 | 0.00 | 0.017872 |
| NV5 | 70-90 | 68.19 | 13.58 | 18.23 | 0.793 | 0.433 | 5.02 | 0.46 | 13.18 | 86.82 | 0.00 | 0.018384 |
| AL1 | 0-10 | 76.88 | 9.41 | 13.71 | 0.883 | 0.557 | 8.17 | 3.84 | 18.02 | 81.86 | 0.13 | 0.010161 |
| AL1 | 10-20 | 76.04 | 9.18 | 14.78 | 0.873 | 0.538 | 8.28 | 1.47 | 19.71 | 80.29 | 0.00 | 0.012138 |
| AL1 | 20-30 | 69.58 | 10.61 | 19.81 | 0.804 | 0.433 | 6.56 | 1.09 | 20.86 | 79.14 | 0.00 | 0.014702 |
| AL1 | 30-50 | 66.87 | 10.95 | 22.18 | 0.775 | 0.393 | 6.11 | 0.97 | 20.25 | 79.75 | 0.00 | 0.015563 |
| AL1 | 50-70 | 67.25 | 8.83 | 23.92 | 0.776 | 0.384 | 7.62 | 0.68 | 23.08 | 76.92 | 0.00 | 0.016707 |
| AL1 | 70-90 | 61.07 | 9.72 | 29.21 | 0.709 | 0.310 | 6.28 | 0.65 | 23.57 | 76.43 | 0.00 | 0.016748 |
| AL2 | 0-10 | 79.71 | 8.96 | 11.33 | 0.913 | 0.615 | 8.90 | 2.87 | 21.89 | 77.83 | 0.28 | 0.00982 |
| AL2 | 10-20 | 78.54 | 8.91 | 12.55 | 0.900 | 0.588 | 8.81 | 1.63 | 21.38 | 78.62 | 0.00 | 0.011223 |
| AL2 | 20-30 | 74.69 | 10.82 | 14.49 | 0.860 | 0.526 | 6.90 | 1.67 | 20.21 | 79.79 | 0.00 | 0.011167 |
| AL2 | 30-50 | 77.62 | 8.24 | 14.14 | 0.890 | 0.561 | 9.43 | 1.50 | 21.90 | 78.10 | 0.00 | 0.011778 |
| AL2 | 50-70 | 70.23 | 15.40 | 14.38 | 0.817 | 0.482 | 4.56 | 0.95 | 19.96 | 80.04 | 0.00 | 0.015731 |
| AL2 | 70-90 | 65.27 | 16.96 | 17.77 | 0.765 | 0.411 | 3.85 | 0.77 | 19.27 | 80.73 | 0.00 | 0.016818 |
| AL3 | 0-10 | 72.21 | 12.30 | 15.50 | 0.835 | 0.492 | 5.87 | 5.13 | 17.42 | 82.55 | 0.04 | 0.01022 |
| AL3 | 10-20 | 76.67 | 12.18 | 11.15 | 0.884 | 0.580 | 6.29 | 4.27 | 18.73 | 81.27 | 0.00 | 0.010108 |
| AL3 | 20-30 | 78.26 | 9.56 | 12.18 | 0.898 | 0.588 | 8.19 | 4.13 | 19.09 | 80.91 | 0.00 | 0.010076 |
| AL3 | 30-50 | 83.37 | 8.82 | 7.81 | 0.953 | 0.703 | 9.45 | 3.98 | 17.97 | 82.03 | 0.00 | 0.010175 |
| AL3 | 50-70 | 82.93 | 8.70 | 8.37 | 0.948 | 0.690 | 9.53 | 2.02 | 11.61 | 88.39 | 0.00 | 0.011131 |
| AL4 | 0-10 | 80.81 | 7.11 | 12.08 | 0.923 | 0.620 | 11.37 | 3.20 | 17.96 | 82.04 | 0.00 | 0.010183 |
| AL4 | 10-20 | 84.53 | 5.61 | 9.85 | 0.962 | 0.695 | 15.05 | 2.44 | 17.59 | 82.26 | 0.15 | 0.010297 |
| AL4 | 20-30 | 77.43 | 9.88 | 12.69 | 0.889 | 0.573 | 7.84 | 1.88 | 16.47 | 83.53 | 0.00 | 0.010947 |
| AL4 | 30-50 | 75.37 | 9.19 | 15.44 | 0.866 | 0.525 | 8.21 | 1.37 | 16.22 | 83.78 | 0.00 | 0.013123 |
| AL4 | 50-70 | 72.37 | 14.15 | 13.49 | 0.839 | 0.511 | 5.12 | 1.01 | 16.58 | 83.42 | 0.00 | 0.015701 |
| AL5 | 0-10 | 82.91 | 8.02 | 9.07 | 0.947 | 0.682 | 10.34 | 3.71 | 16.66 | 81.68 | 1.66 | 0.010154 |
| AL5 | 10-20 | 80.79 | 6.76 | 12.46 | 0.922 | 0.616 | 11.96 | 3.36 | 19.44 | 80.09 | 0.47 | 0.010007 |
| AL5 | 20-30 | 80.09 | 7.90 | 12.02 | 0.916 | 0.612 | 10.14 | 2.74 | 18.59 | 81.41 | 0.00 | 0.010157 |
| AL5 | 30-50 | 70.36 | 10.94 | 18.70 | 0.813 | 0.448 | 6.43 | 1.82 | 18.28 | 81.72 | 0.00 | 0.010906 |
| AL5 | 50-70 | 26.45 | 12.67 | 60.88 | 0.333 | 0.089 | 2.09 | 0.33 | 18.69 | 81.31 | 0.00 | 0.01793 |
| CL1 | 0-10 | 74.15 | 10.58 | 15.26 | 0.854 | 0.513 | 7.01 | 2.47 | 18.69 | 81.32 | 0.00 | 0.010202 |
| CL1 | 10-20 | 74.09 | 11.13 | 14.78 | 0.854 | 0.517 | 6.66 | 2.29 | 21.14 | 78.87 | 0.00 | 0.010058 |
| CL1 | 20-30 | 61.30 | 16.93 | 21.77 | 0.721 | 0.354 | 3.62 | 1.64 | 19.29 | 80.71 | 0.00 | 0.011375 |
| CL2 | 0-10 | 66.82 | 11.88 | 21.30 | 0.775 | 0.399 | 5.63 | 2.47 | 23.03 | 76.60 | 0.37 | 0.009769 |
| CL2 | 10-20 | 66.52 | 12.26 | 21.22 | 0.773 | 0.397 | 5.43 | 1.94 | 24.57 | 75.10 | 0.33 | 0.010042 |
| CL2 | 20-30 | 66.96 | 13.94 | 19.10 | 0.780 | 0.416 | 4.80 | 1.28 | 21.36 | 78.64 | 0.00 | 0.013215 |
| CL2 | 30-50 | 64.19 | 17.74 | 18.07 | 0.754 | 0.400 | 3.62 | 0.95 | 21.09 | 78.91 | 0.00 | 0.015642 |
| CL2 | 50-70 | 52.47 | 23.84 | 23.69 | 0.633 | 0.287 | 2.20 | 0.99 | 23.21 | 76.79 | 0.00 | 0.015166 |
| CL3 | 0-10 | 66.20 | 16.52 | 17.28 | 0.774 | 0.423 | 4.01 | 4.40 | 18.07 | 81.93 | 0.00 | 0.010166 |
| CL3 | 10-20 | 68.33 | 18.37 | 13.30 | 0.800 | 0.473 | 3.72 | 4.55 | 16.71 | 83.25 | 0.03 | 0.010282 |
| CL3 | 20-30 | 58.27 | 20.78 | 20.95 | 0.693 | 0.338 | 2.80 | 4.69 | 16.45 | 83.49 | 0.06 | 0.010303 |
| CL3 | 30-50 | 84.54 | 7.93 | 7.53 | 0.965 | 0.724 | 10.66 | 5.33 | 17.93 | 82.07 | 0.00 | 0.010178 |
| CL4 | 0-10 | 69.32 | 14.13 | 16.55 | 0.806 | 0.456 | 4.91 | 4.10 | 22.33 | 77.67 | 0.00 | 0.009784 |
| CL4 | 10-20 | 85.94 | 6.52 | 7.54 | 0.979 | 0.744 | 13.18 | 3.99 | 21.32 | 78.68 | 0.00 | 0.009877 |
| CL4 | 20-30 | 80.81 | 8.57 | 10.63 | 0.925 | 0.636 | 9.43 | 4.25 | 19.74 | 80.26 | 0.00 | 0.010018 |
| CL4 | 30-50 | 77.82 | 9.48 | 12.69 | 0.893 | 0.578 | 8.21 | 2.85 | 19.65 | 80.35 | 0.00 | 0.010051 |
| CL5 | 0-10 | 52.93 | 14.66 | 32.41 | 0.626 | 0.249 | 3.61 | 2.54 | 22.16 | 77.84 | 0.00 | 0.00987 |
| CL5 | 10-20 | 52.33 | 23.84 | 23.83 | 0.631 | 0.285 | 2.20 | 1.06 | 19.20 | 80.80 | 0.00 | 0.015103 |
| CL5 | 20-30 | 51.42 | 22.81 | 25.77 | 0.620 | 0.271 | 2.25 | 0.89 | 17.85 | 82.11 | 0.03 | 0.016322 |
| CL6 | 0-10 | 57.24 | 16.36 | 26.40 | 0.676 | 0.301 | 3.50 | 3.36 | 17.66 | 82.34 | 0.00 | 0.010206 |
| CL6 | 10-20 | 76.19 | 9.22 | 14.59 | 0.875 | 0.541 | 8.27 | 2.95 | 18.53 | 81.47 | 0.00 | 0.010144 |
| CL6 | 20-30 | 72.41 | 12.36 | 15.22 | 0.837 | 0.496 | 5.86 | 3.33 | 17.19 | 82.81 | 0.00 | 0.010248 |
| CL6 | 30-50 | 80.07 | 11.07 | 8.86 | 0.920 | 0.647 | 7.23 | 2.82 | 18.45 | 81.55 | 0.00 | 0.01016 |
| CL7 | 0-10 | 53.34 | 16.52 | 30.13 | 0.633 | 0.261 | 3.23 | 2.06 | 20.90 | 79.10 | 0.00 | 0.010264 |
| CL7 | 10-20 | 35.15 | 22.77 | 42.08 | 0.441 | 0.147 | 1.54 | 0.50 | 20.64 | 79.36 | 0.00 | 0.017465 |
| CL7 | 20-30 | 23.24 | 15.88 | 60.89 | 0.302 | 0.084 | 1.46 | 0.46 | 20.04 | 79.91 | 0.05 | 0.017595 |
| CL7 | 30-50 | 35.28 | 20.24 | 44.48 | 0.439 | 0.142 | 1.74 | 0.43 | 19.17 | 80.83 | 0.00 | 0.017756 |
| CL7 | 50-70 | 64.40 | 17.28 | 18.32 | 0.756 | 0.400 | 3.73 | 0.55 | 18.72 | 81.28 | 0.00 | 0.017585 |
| CL8 | 0-10 | 63.21 | 19.27 | 17.52 | 0.745 | 0.396 | 3.28 | 4.78 | 15.85 | 84.09 | 0.06 | 0.010355 |
| CL8 | 10-20 | 71.43 | 15.76 | 12.81 | 0.831 | 0.507 | 4.53 | 4.58 | 16.45 | 83.55 | 0.00 | 0.010307 |
| CL8 | 20-30 | 84.86 | 8.10 | 7.04 | 0.969 | 0.735 | 10.48 | 5.34 | 16.44 | 83.57 | 0.00 | 0.010308 |
| CL8 | 30-50 | 85.24 | 7.44 | 7.32 | 0.972 | 0.737 | 11.46 | 3.65 | 14.75 | 85.25 | 0.00 | 0.010455 |
| CL8 | 50-70 | 84.81 | 8.24 | 6.95 | 0.968 | 0.735 | 10.30 | 2.40 | 13.61 | 86.39 | 0.00 | 0.010668 |
| Min | | 23.24 | 4.65 | 3.95 | 0.302 | 0.084 | 1.46 | 0.33 | 11.61 | 75.10 | 0.00 | 0.009558 |
| Max | | 90.83 | 23.84 | 60.89 | 1.030 | 0.868 | 19.53 | 5.80 | 24.57 | 88.39 | 3.83 | 0.018384 |
| Average | | 71.76 | 11.53 | 16.71 | 0.829 | 0.516 | 7.47 | 2.64 | 18.67 | 81.22 | 0.12 | 0.011946 |
